# Safety of Sabin-derived inactivated poliovirus vaccine in adult booster recipients: a systematic review and meta-analysis

**DOI:** 10.3389/fpubh.2026.1883550

**Published:** 2026-09-03

**Authors:** Muhammad Hussain, Muhammad Hassan, Abdul Hannan, Arham Kamil, Muhammad Tayyab Azam, Zain Ul Abideen Shahid, Hasibullah Aminpoor, Suhaylah Gore, Faizan Ahmed, Anna Guseva

**Affiliations:** 1 Faculty of Medicine, Dow University of Health Sciences, Karachi, Pakistan; 2 Faculty of Medicine, Kabul University of Medical Sciences “Abu Ali Ibn Sina”, Kabul, Afghanistan; 3 Saint Georges University Medical School, St. George's, Grenada; 4 Department of Internal Medicine, Jersey Shore University Medical Center, Neptune, NJ, United States; 5 Russian University of Medicine, Moscow, Russia

**Keywords:** metanalysis, polio, public health, safety, vaccine

## Abstract

**Background:**

Poliomyelitis remains a public health concern in select regions. Sabin strain-derived inactivated poliovirus vaccine (sIPV) offers a potentially safer and cost-effective alternative to conventional IPV (cIPV) for adult booster immunization.

**Objective:**

To systematically review and meta-analyze evidence on the safety of sIPV in adults.

**Methods:**

We searched PubMed, Embase, Cochrane, and Scopus through January 2026 for randomized controlled trials comparing sIPV with cIPV/w-IPV or placebo in adults. Safety outcomes included local and systemic adverse events: pain, erythema, induration, fever, headache, malaise, and allergic reactions. Data extraction and risk of bias assessment were independently performed. Meta-analysis was conducted using random-effects models in RevMan 5.4, reporting risk ratios (RR) with 95% confidence intervals (CI). Evidence certainty was assessed using GRADE.

**Results:**

Four trials encompassing 70–340 adults were included. Pooled analyses demonstrated no statistically significant differences in any adverse events between sIPV and cIPV: pain (RR 1.57, 95% CI 0.98–2.50), erythema (RR 1.54, 95% CI 0.64–3.69), induration (RR 0.33, 95% CI 0.04–3.06), fever (RR 0.96, 95% CI 0.39–2.33), headache (RR 0.70, 95% CI 0.21–2.37), and malaise (RR 0.75, 95% CI 0.17–3.27). Heterogeneity was negligible (*I*^2^ = 0%). Overall, adverse events were mild, transient, and comparable across vaccines and most included trials reported no serious adverse events (SAEs), indicating a favorable safety profile. Certainty of evidence was low to very low due to small sample sizes and imprecision.

**Conclusion:**

sIPV demonstrates a safety profile comparable to conventional IPV in adult booster recipients, supporting its use as a viable alternative in polio eradication strategies. Further large-scale and long-term studies are warranted to confirm these findings.

**Systematic Review Registration:**

identifier: CRD420261329648.

## Introduction

Poliomyelitis is an acute viral disease that primarily affects motor neurons of the central nervous system and most commonly occurs in childhood. Introduction of both inactivated poliovirus vaccine (IPV) and oral poliovirus vaccine (OPV) has resulted in the near worldwide eradication of the disease, except in a few countries, namely Afghanistan and Pakistan. Strains of wild type 2 and 3 have been eradicated (1, 2), but type 1 strains persist.

Additionally, vaccine-derived polioviruses have also emerged and are associated with OPV use (3). These strains develop when viruses undergo mutations that confer neurovirulence during intestinal shedding (3, 4). Other times, these mutations can cause vaccine-associated paralytic poliomyelitis, which can spread, albeit to a limited extent, affecting mostly close contacts (3).

Therefore, to achieve complete polio eradication and reduce the risk of adverse events associated with OPV, greater emphasis is being placed on shifting toward IPV-based immunization. However, given the comparatively higher production cost of IPV compared with OPV, the development of cost-effective IPV alternatives is imperative. In this regard, Sabin strain-derived IPV (sIPV) represents a viable option (5). Phase I/II trials have evaluated Sabin-IPV as boosters in previously vaccinated adults, but no systematic review or meta-analysis has synthesized evidence on its safety compared to conventional IPV.

Accordingly, we conducted a systematic review and meta-analysis of published studies on the safety of sIPV in adults to address this gap and provide evidence to inform clinicians and policymakers.

## Method

This systematic review and meta-analysis was conducted according to the preferred Reporting Items for Systematic Reviews and Meta-Analyses (PRISMA) guidelines (6). The protocol was registered in the International Prospective Register of Systematic Reviews database (PROSPERO 2026 CRD420261329648).

## Data sources and search strategy

A systematic search was performed across PubMed, Embase, Cochrane, and Scopus. The final search was performed on 15 January 2026. The strategy used free-text keywords along with MeSH terms (Medical Subject Headings). A combination of several keywords was used, such as “Sabin inactivated poliovirus vaccine,” “sIPV,” “polio booster,” and “adult vaccination” as intervention. Studies across the time periods were included, as no limit was applied on publication date and o nly studies published in English were included. No additional search strategies were utilized. The full search Strategy is presented in Supplementary Table 1.

## Eligibility criteria and study selection

Trials were included if they met the following criteria: (1) randomized controlled trials (phase 1–3), (2) adult participants receiving a booster polio vaccine, (3) comparison of sIPV vs. conventional IPV/wIPV or placebo, (4) reported safety outcomes (local or systemic adverse events). Studies were excluded if they (1) did not report safety outcomes, (2) were pediatric studies, or (3) were non-randomized trials, observational studies or case reports. Screening of included articles was performed by Rayyan AI (7) to remove duplicates and then manual screening to exclude trials that did not meet the inclusion criteria. Two independent reviewers screened all titles and abstracts for relevance, and trials that met the criteria were opted for full-text screening; any disagreements were resolved by consensus or by consultation with an independent reviewer. Full-texts were reviewed by one independent reviewer followed by a second independent reviewer for eligibility according to predefined inclusion criteria, and any further disagreements were resolved by consensus or by consultation with a third reviewer. The reasons for excluding trials were also recorded.

## Data extraction and outcome of interest

Two independent reviewers, using a standardized data extraction sheet, extracted the following baseline and study characteristics: Author/Year, Journal, DOI, Clinical Trial Registration, and Study Type (Phase I/II/III). Followed Patient demographics (sample size), Treatment regimen (dose, frequency) and Primary Endpoint(s) reported by the studies. The following are the outcomes of interest: pain, erythema, induration, fever, malaise, headache, and other reported adverse events, along with serious adverse events. Serious adverse events (SAEs) were defined according to the International Council for Harmonization (ICH) guidelines as events that resulted in death or life-threatening events such as inpatient hospitalization or prolongation of hospitalization, a persistent or significant disability, or any congenital defect. For all outcomes, the number of participants experiencing each adverse event and the total number of participants were extracted for each cohort from each study and used for the meta-analysis. The extracted data was verified by another independent reviewer.

## Risk of bias and quality assessment

The Risk of Bias for randomized trials was evaluated using the Cochrane Risk-of-Bias 2.0 tool (8), which evaluates it across five domains: randomization, deviations from Interventions, missing Outcome Data, Measurement of outcome, and selection of reported result. Each trial received an overall rating of “low risk of bias,” “moderate risk of bias,” “and high risk of bias” and some concerns. The assessments conducted were reviewed by a second reviewer. The summary of risk of bias and quality assessment has been summarized in Figure 2. The overall certainty and confidence in the body of evidence were assessed using the Grading of Recommendations Assessment, Development and Evaluation (GRADE) (9) approach, which evaluates on the basis of the following: (1) risk of bias, (2) inconsistency, (3) indirectness, (4) imprecision, and (5) publication bias.

## Analysis

Adverse event-related outcomes (dichotomous) were analyzed using risk-ratios (RRs) with 95% confidence intervals (CIs). Meta-analytical pooling was performed using a random-effects model based on the DerSimonian–Laird method to account for heterogeneity between studies. The quantitative synthesis was performed in RevMan [Version 5.4] (10). Heterogeneity was quantified using the *I*^2^ statistic with values of 0–25%, 25–50%, 50–75%, and 75–100% indicating Low heterogeneity, Moderate heterogeneity, Substantial heterogeneity, and Considerable heterogeneity, respectively. Publication bias was assessed using funnel plots and Egger's regression test for outcomes with data from three or more studies (*k* ≥ 3). Egger's test was not performed for outcomes informed by fewer than three studies (induration and headache), given the statistical unreliability of the test at very low *k*. Given the overall small number of included studies per outcome, results of publication bias testing were interpreted with caution. This study used data from published literature and required no ethical approval.

## Results

## Study selection

**Figure 1:**
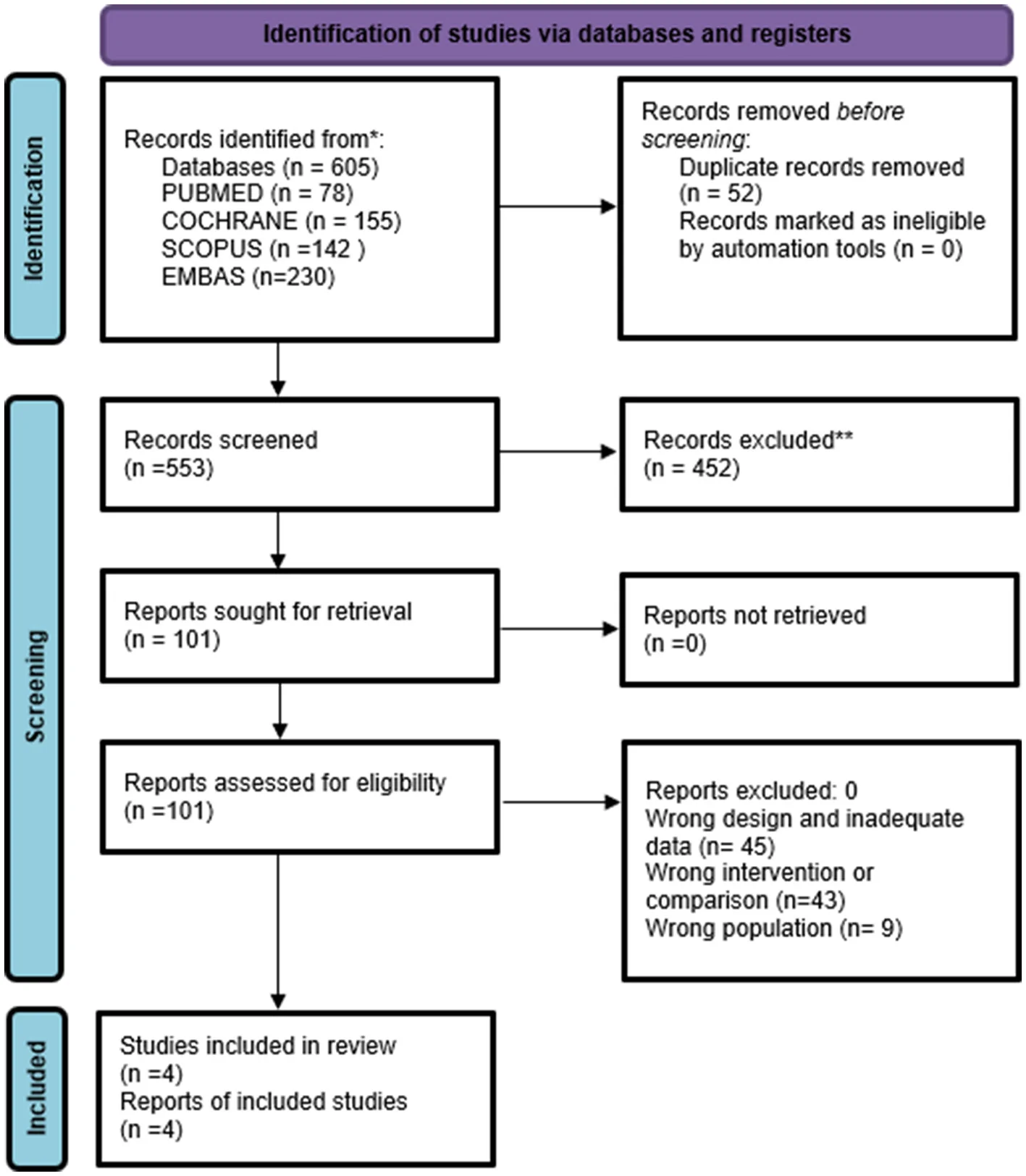
PRISMA flow chart.

A total of 605 records were initially identified through database searching. After removing 52 duplicates, 553 records were screened. Of these, 452 records were excluded based on title and abstract screening. Out of the 101 reports sought for retrieval, all 101 were assessed for eligibility. Ultimately, 4 studies met the inclusion criteria and were included in this systematic review and meta-analysis (Figure 1).

## Study and patient characteristics

A total of four studies (11–14) conducted in Panama, Cuba, the Netherlands, and Russia were included. The sample size per study arm ranged from 15 to 30 participants.

The mean age of participants ranged from 21 ± 2 years to 37.4 ± 11.4 years. Sex distribution was reported in Cramer et al. (4/16 vs. 7/13) and Piniaeva et al. (14/16 vs. 19/11), while it was not reported in the remaining studies. Baseline seropositivity was reported in Resik et al. (80–100%) and Verdijk et al. (73–100%) but not reported in Cramer et al. and Piniaeva et al. (Table 1).

**Table 1:** Characteristics of included studies.

Study (Author et al.)	Study location	Group	*N* (per group)	Mean Age (years ±SD)	Sex (Male/Female)	Baseline seropositivity
Cramer et al.	Hospital del Niño Doctor José Renán Esquivel, Panama City, Panama	High-dose Sabin inactivated poliovirus vaccine	20	34.9 ± 8.56	4/16	Not reported
Cramer et al.	Hospital del Niño Doctor José Renán Esquivel, Panama City, Panama	Placebo	20	35.1 ± 8.23	7/13	Not reported
Resik et al.	Havana and Camagüey, Cuba	Sabin inactivated poliovirus vaccine	20	21 ± 2	Not reported	Type 1: 95%, Type 2: 90%, Type 3: 90%
Resik et al.	Havana and Camagüey, Cuba	Salk inactivated poliovirus vaccine	20	21 ± 2	Not reported	Type 1: 80%, Type 2: 100%, Type 3: 85%
Verdijk et al.	Bilthoven, The Netherlands	Sabin inactivated poliovirus vaccine	15	33.8 ± 8.8	Not reported	Sabin type 1: 87%, Sabin type 2: 93%, Sabin type 3: 73%
Verdijk et al.	Bilthoven, The Netherlands	Conventional (Salk) inactivated poliovirus vaccine	15	31.1 ± 9.1	Not reported	Sabin type 1: 93%, Sabin type 2: 100%, Sabin type 3: 80%
Piniaeva et al.	Moscow and Perm, Russia	PoliovacSin	30	37.4 ± 11.4	14/16	Not reported
Piniaeva et al.	Moscow and Perm, Russia	Placebo	30	32.5 ± 11.4	19/11	Not reported

## Any pain

**Figure 2:**
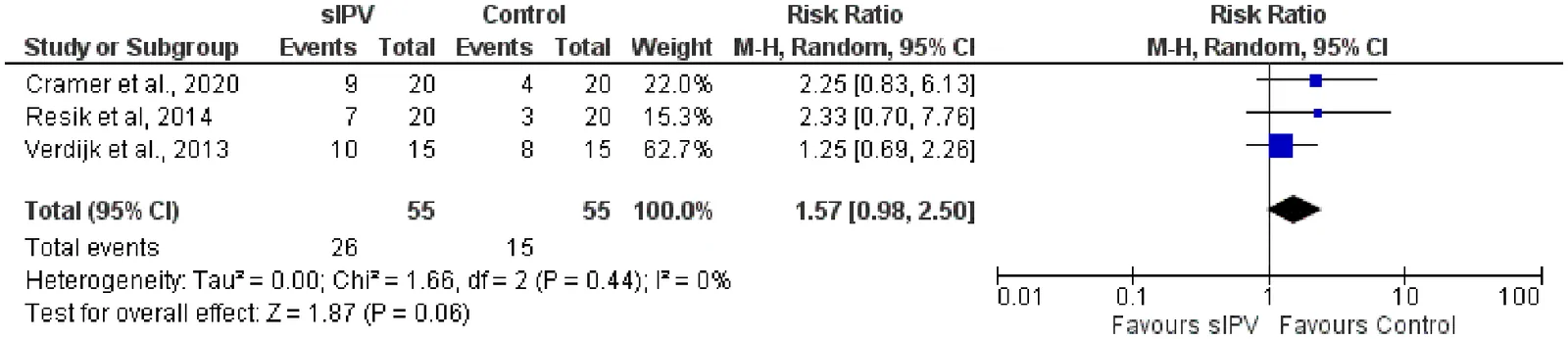
Any pain, sIPV vs. control (RR).

Three studies evaluated the presence of pain in both the sIPV and control groups. The pooled analysis demonstrated no statistically significant difference in any sort of pain with sIPV with an overall risk ratio (RR) of 1.57 (95% CI: 0.98–2.50, *I*^2^ = 0%, *p* = 0.06) (Figure 2).

## Erythema

**Figure 3:**
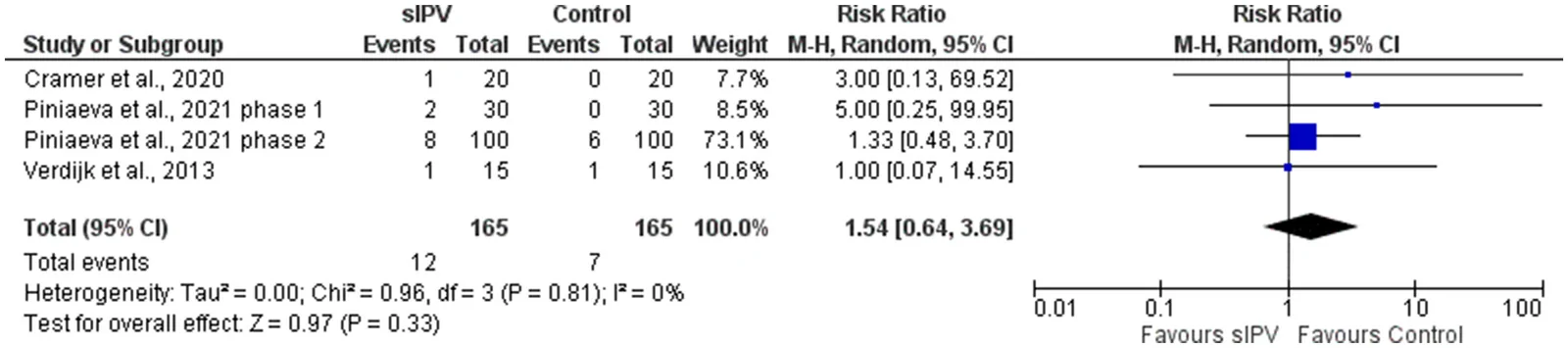
Erythema, sIPV vs. control (RR).

Four studies evaluated the presence of erythema in both the sIPV and control groups. The pooled analysis demonstrated no statistically significant difference in erythema with sIPV with an overall risk ratio (RR) of 1.54 (95% CI: 0.64–3.69, *I*^2^ = 0%, *p* = 0.33) (Figure 3).

## Induration

**Figure 4:**
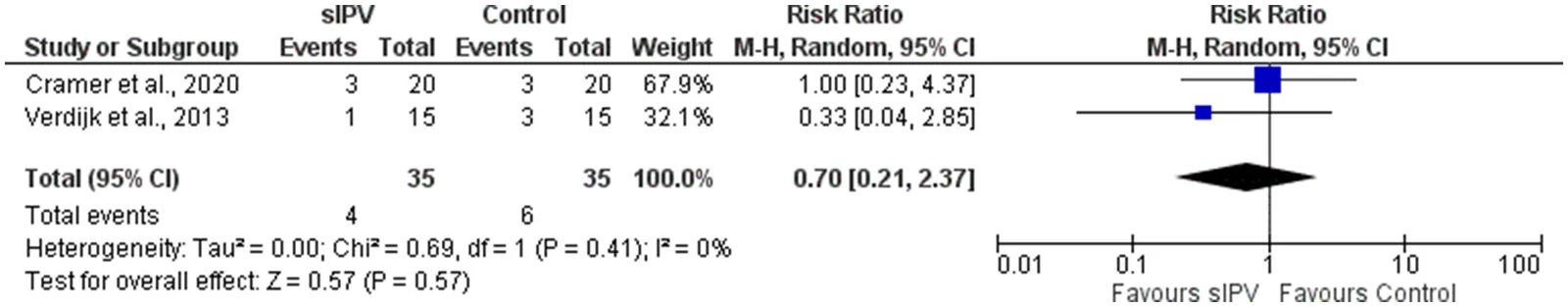
Induration, sIPV vs. control (RR).

Two studies evaluated the induration in both the sIPV and control groups. The pooled analysis demonstrated no statistically significant difference in induration with sIPV with an overall risk ratio (RR) of 0.33 (95% CI: 0.04–3.06, *I*^2^ = 0%, *p* = 0.33) (Figure 4).

## Fever

**Figure 5:**
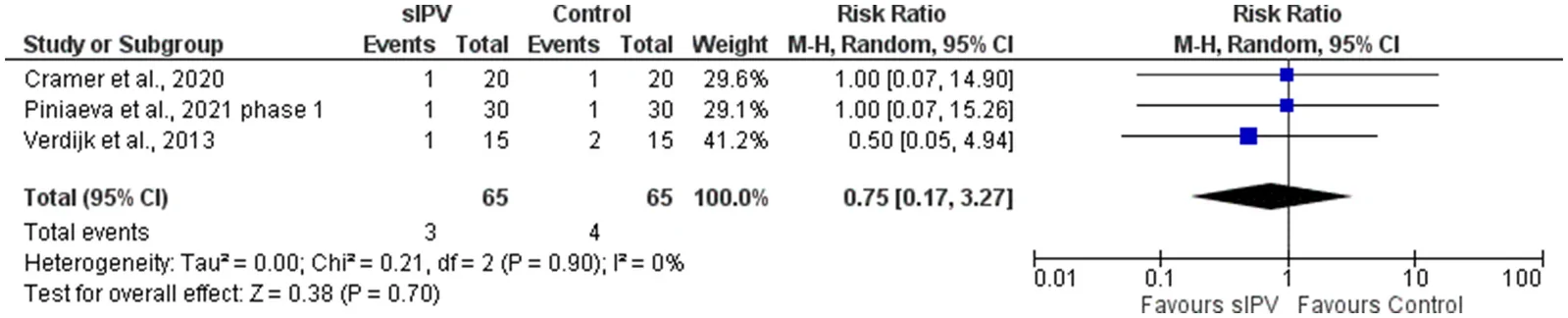
Fever, sIPV vs. control (RR).

Four studies evaluated the presence of fever in both the sIPV and control groups. The pooled analysis demonstrated no statistically significant difference in fever with sIPV with an overall risk ratio (RR) of 0.96 (95% CI: 0.39–2.33, *I*^2^ = 0%, *p* = 0.92) (Figure 5).

## Headache

**Figure 6:**
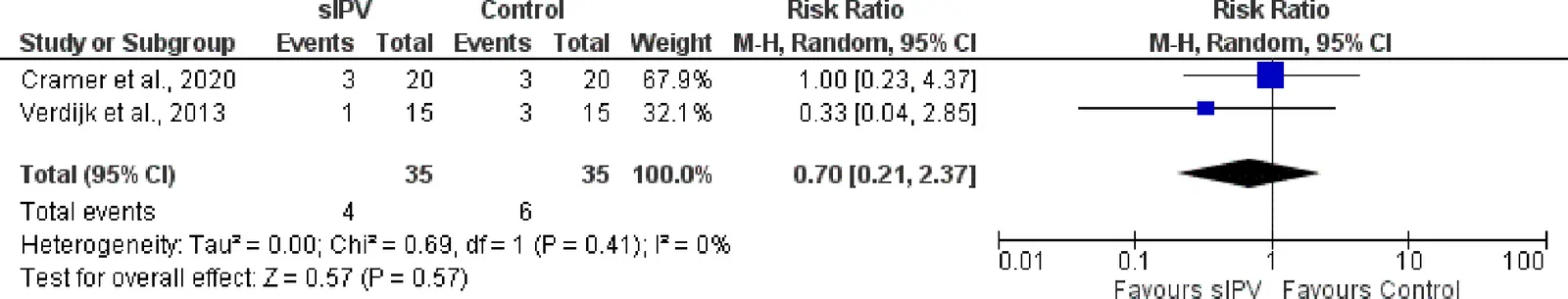
Headache, sIPV vs. control (RR).

Two studies evaluated headaches in both the sIPV and control groups. The pooled analysis demonstrated no statistically significant difference in headaches with sIPV with an overall risk ratio (RR) of 0.70 (95% CI: 0.21–2.37, *I*^2^ = 0%, *p* = 0.57) (Figure 6).

## Malaise

Three studies evaluated the presence of malaise in both the sIPV and control groups. The pooled analysis demonstrated no statistically significant difference in malaise with sIPV with an overall risk ratio (RR) of 0.75 (95% CI: 0.17–3.27, *I*^2^ = 0%, *p* = 0.70) (Supplementary Figure 1).

## Moderate Pain

Three studies evaluated the presence of moderate pain in both the sIPV and control groups. The pooled analysis demonstrated no statistically significant difference in moderate pain with sIPV with an overall risk ratio (RR) of 3.28 (95% CI: 0.69–15.53, *I*^2^ = 0%, *p* = 0.14) (Supplementary Figure 2).

## Mild pain

Three studies evaluated the presence of mild pain in both the sIPV and control groups. The pooled analysis demonstrated no statistically significant difference in mild pain with sIPV with an overall risk ratio (RR) of 1.98 (95% CI: 0.90–4.40, *I*^2^ = 0%, *p* = 0.09) (Supplementary Figure 3).

## Serious adverse events (SAEs)

The majority of the included trials reported no serious adverse events.

## Sensitivity analysis

Leave-one-out sensitivity analyses demonstrated no significant change in findings upon exclusion of any single study. All analyses showed low heterogeneity (*I*^2^ = 0%) and reinforced the overall safety profile of sIPV, with most local and systemic reactions comparable to control. For moderate pain, there was a non-significant increase with sIPV (RR 5.00, 95% CI [0.60, 41.69]; *p* = 0.14) (Supplementary Figure 4). Malaise showed no difference (RR 1.00, 95% CI [0.15, 6.81]; *p* = 1.00) (Supplementary Figure 5). Similarly, fever rates were comparable between groups (RR 1.05, 95% CI [0.41, 2.65]; *p* = 0.92) (Supplementary Figure 6). Erythema exhibited a non-significant elevation with sIPV (RR 1.62, 95% CI [0.64, 4.09]; *p* = 0.31) (Supplementary Figure 7). In contrast, any pain remained significantly higher in the sIPV arm (RR 2.28, 95% CI [1.06, 4.93]; *p* = 0.04) (Supplementary Figure 8).

## Publication bias

Publication bias was assessed via visual inspection of funnel plots and Egger's regression test. Egger's test could not be performed for induration and headache due to insufficient studies (*k* < 3). Among evaluable outcomes, statistically significant intercepts were observed for malaise (*p* = 0.038), moderate pain (*p* = 0.001), and fever (*p* < 0.001); however, the extreme t-statistics for moderate pain and fever reflect near-zero standard error variance across included studies rather than true funnel asymmetry, and should not be interpreted as evidence of publication bias. No significant asymmetry was detected for any pain (*p* = 0.788), erythema (*p* = 0.922), or mild pain (*p* = 0.527). Overall, given that all outcomes were informed by only two to four studies, Egger's test was substantially underpowered across the board, and definitive conclusions regarding publication bias cannot be drawn from these analyses (Supplementary Figure 9).

## Quality assessment

**Figure 7:**
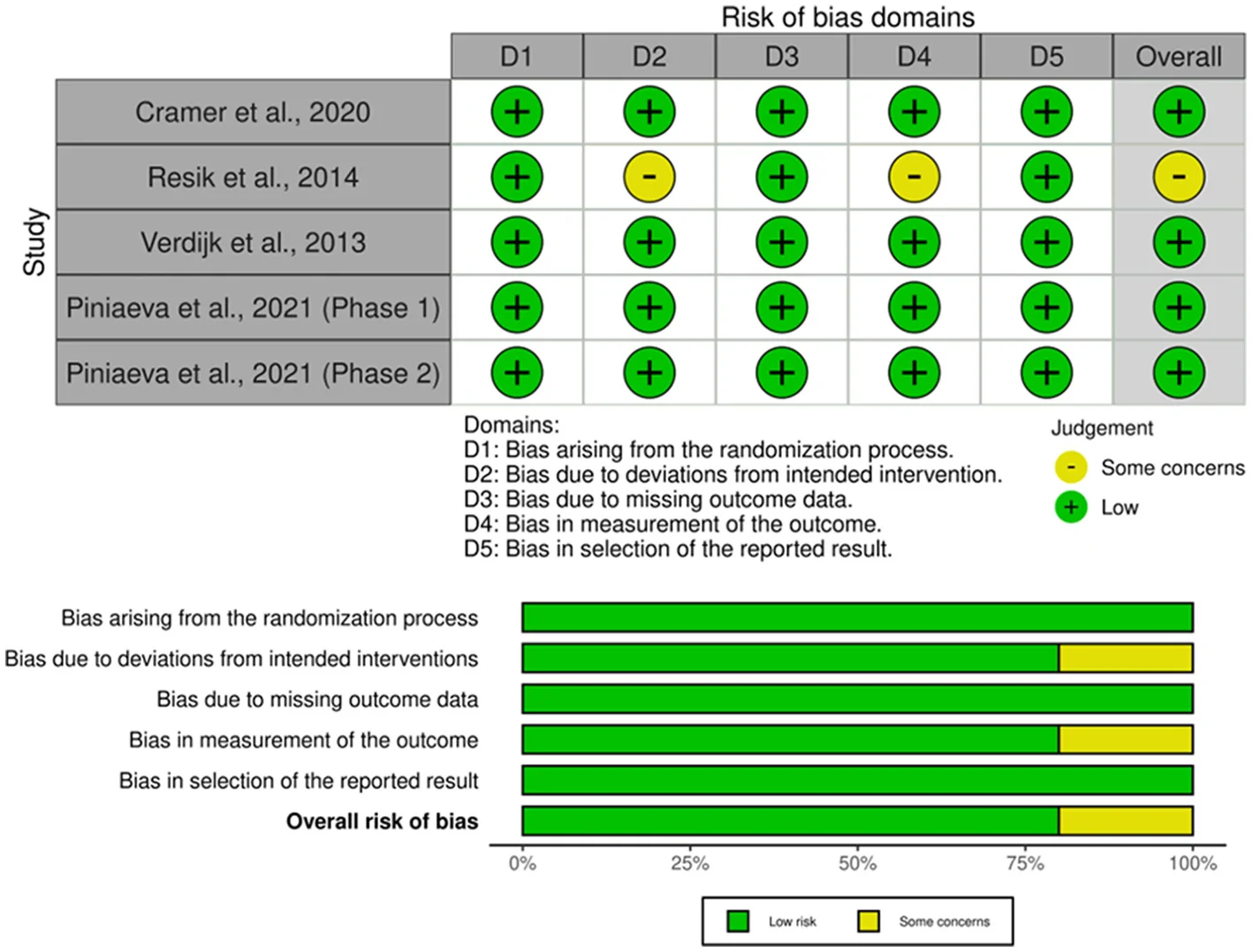
Risk of bias assessment of included studies.

The risk of bias assessment indicated that three included randomized controlled trials were judged to have an overall low risk of bias across all assessed domains (D1–D5). In contrast, the study by Resik et al. (13) was rated as having some concerns overall, primarily due to concerns identified in Domain 2 and Domain 4, while the remaining domains were assessed as low risk. Overall, the included studies demonstrated acceptable methodological quality (Figure 7).

## Certainty of evidence

The evidence on the safety of the polio vaccine in adults, drawn from 2 to 4 studies per outcome with 70–340 participants, suggests that the vaccine is generally well-tolerated, with most adverse events being mild and transient. Outcomes such as pain, erythema, induration, fever, headache, and malaise occurred with variable frequency across studies. The certainty of evidence was low to very low for most outcomes, primarily due to serious imprecision reflected in wide confidence intervals and, in some cases, small sample sizes. Minor indirectness was noted for certain outcomes. Overall, while the polio vaccine appears safe in adults, the limited number of studies reduces confidence in the precision of these estimates (Supplementary Table 2).

## Discussion

This systematic review and meta-analysis synthesized the available randomized evidence comparing the safety of Sabin-derived inactivated poliovirus vaccine (sIPV) with conventional IPV when administered as booster doses in previously immunized adults. Across all evaluated local and systemic adverse events, including pain, erythema, induration, fever, headache, and malaise, no statistically significant differences were observed between the two vaccine platforms. Overall, these findings suggest that sIPV has a generally comparable short-term reactogenicity profile to conventional IPV in adult booster recipients, while recognizing the limitations of the available evidence.

The absence of significant differences in local reactogenicity responses is biologically plausible. Both s-IPV and conventional IPV are derived from formalin-inactivated poliovirus antigens and are therefore not replication competent and restricted to antigen induced immune responses only (5). In adults with prior IPV or OPV immunity, a booster response will primarily stimulate a quick anamnestic humoral immune response through memory B cells and not elicit significant primary innate inflammatory responses (15). This immunological setting inherently restricts systemic reactogenicity and may explain the attenuation of any existing differences in reactogenicity between different seed strains of poliovirus. Although genetically distinct from wild-type strains of poliovirus used in conventional IPV production, antigen preservation after inactivation appears sufficient to maintain similar local inflammatory responses (16). The absence of differences in systemic reactogenicity further supports the postulation that differences in seed strain derivation do not impact post-booster inflammatory responses in immunologically primed adults.

Notably, several pooled point estimates, for example, any pain (RR 1.57, 95% CI 0.98–2.50) and mild pain (RR 1.98, 95% CI 0.90–4.40), approached statistical significance but remained imprecise. These wide confidence intervals reflect limited event counts and modest sample sizes rather than definitive evidence of differential reactogenicity. Therefore, although no statistically significant differences were observed between sIPV and conventional IPV, these findings should not be interpreted as evidence of equivalence. The limited sample sizes, low event rates, and wide confidence intervals reduce the precision of the pooled estimates and do not exclude the possibility of clinically meaningful differences. Larger, adequately powered studies are therefore required to more definitively compare the safety profiles of the two vaccine platforms.

Our findings align with the broader literature demonstrating favorable safety profiles for inactivated poliovirus vaccines. Extensive post-licensure surveillance and clinical trials have consistently shown that IPV is associated predominantly with mild, self-limited local reactions and infrequent systemic events (17). Randomized evaluations of Sabin-derived IPV in both pediatric and adult populations have similarly reported acceptable tolerability without excess safety signals (11, 18). However, prior syntheses have largely emphasized immunogenicity outcomes or primary immunization series. By focusing specifically on adult booster recipients, our analysis addresses a distinct and policy relevant population in whom immunological priming alters reactogenicity dynamics and where data have been comparatively limited.

The implications of these findings in the realm of public health are particularly noteworthy in the context of the global polio eradication endgame, wherein the containment of wild poliovirus materials becomes more restrictive, and the diversification of manufacturing processes assumes a position of strategic importance, wherein the use of Sabin-based IPV has a relative biosafety advantage over wild-type-derived production processes. These findings of relative safety in the adult booster population would be particularly advantageous in the era of increased vaccine safety concerns, whereas the era of vaccine hesitancy has led to a need to sustain public confidence through the demonstration of relative safety.

Methodologically, the included trials exhibited strong internal validity, with appropriate randomization, allocation concealment, blinding, and outcome reporting. The application of a random effects model, despite negligible heterogeneity, provides conservative pooled estimates that account for potential underlying differences in study design or population characteristics. Nevertheless, certain constraints merit careful consideration. The total number of trials and participants was limited, reducing the capacity to detect rare or delayed adverse events. Analyses were based on aggregate data, precluding patient level exploration of effect modification by age, baseline immunity, comorbidity, or prior vaccine exposure. Follow-up periods were short and focused primarily on early reactogenicity, limiting inference regarding longer term safety. Furthermore, no large post-marketing surveillance studies evaluating the safety of Sabin-derived IPV in adult populations are currently available. Although randomized controlled trials provide high-quality evidence under controlled conditions, they are generally not designed to detect rare or delayed adverse events that may emerge following widespread vaccine use. Consequently, the current evidence base should be interpreted with caution, and continued pharmacovigilance together with large post-licensure safety studies will be important to further characterize the long-term safety profile of sIPV in adults. Additionally, with few studies available, formal assessment of publication bias is inherently underpowered, and small study effects cannot be excluded.

Future studies should also extend beyond the controlled trial setting, including large post marketing surveillance studies that can detect unusual side effects, as well as pragmatic studies that can include more diverse populations of adults. Individual participant data meta-analysis could also provide more sophisticated subgroup analyses, as well as more statistical precision. The inclusion of immunogenicity and reactogenicity could also shed light on the possibility that minor differences between manufacturing processes may impact broader immune-biologic effects. This will be critical as the methods for eradication continue to evolve and the diversity of the IPV vaccine platforms increases.

## Limitations

This meta-analysis has several important limitations. The small number of included trials (*n* = 4) and limited sample sizes (70–340 participants total) substantially reduced statistical power to detect rare adverse events or subtle differences between vaccine formulations. Moreover, the consistently low *I*^2^ values should be interpreted cautiously, as heterogeneity estimates may be unreliable when only a small number of studies are included. Reliance on aggregate-level data precluded exploration of effect modification by participant characteristics such as age, sex, baseline immunity, or comorbidity. Short follow-up periods limited assessment of delayed or long-term adverse events, while incomplete demographic reporting in some trials introduced potential unassessed heterogeneity. The grading of evidence certainty as low to very low, driven by serious imprecision and small sample sizes, limits conclusions regarding absolute equivalence between sIPV and conventional IPV safety profiles. Finally, with only four studies, publication bias and small-study effects cannot be confidently excluded.

## Conclusion

In summary, the available evidence from four randomized controlled trials suggests that Sabin-derived inactivated poliovirus vaccine (sIPV) is generally well tolerated when administered as a booster dose in previously immunized adults, with no statistically significant differences in the evaluated local or systemic adverse events compared with conventional IPV. However, the certainty of the evidence was low to very low for most outcomes because of the limited number of studies, small sample sizes, and imprecision of the pooled estimates. Consequently, these findings should be interpreted with caution and should not be considered definitive evidence of equivalent safety profiles. Additional large, well-designed clinical trials and post-marketing safety studies are needed to further establish the long-term safety of sIPV in adult populations.

## Data Availability

The original contributions presented in the study are included in the article/Supplementary material, further inquiries can be directed to the corresponding author.
