# Supplementary material for: Safety of Sabin-derived inactivated poliovirus vaccine in adult booster recipients: a systematic review and meta-analysis
Source: Front Public Health. 2026 Sep 3;14:1883550. doi: 10.3389/fpubh.2026.1883550 (PMC13582391; doi:10.3389/fpubh.2026.1883550)
Supplement: Supplementary file 1 [file Supplementary_file_1.docx]

**Supplementary Material**

**Supplementary Figure 1.** Malaise, sIPV vs control (RR).

**Supplementary Figure 2.** Moderate pain, sIPV vs control (RR).

**Supplementary Figure 3.** Mild pain, sIPV vs control (RR).

**Supplementary Figure 4.** Sensitivity analysis for moderate pain, sIPV vs control (RR).

**Supplementary Figure 5.** Sensitivity analysis for malaise, sIPV vs control (RR).

**Supplementary Figure 6.** Sensitivity analysis for fever, sIPV vs control (RR).

**Supplementary Figure 7.** Sensitivity analysis for erythema, sIPV vs control (RR).

**Supplementary Figure 8.** Sensitivity analysis for any pain, sIPV vs control (RR).

**Supplementary Figure 9.** Funnel plot assessing publication bias for all outcomes.

| **Database** | **Search Strategy** | **Fields Used** | **Results** |
| --- | --- | --- | --- |
| **PubMed** | **("Poliomyelitis Vaccines"[Mesh] OR "inactivated poliovirus vaccine" OR IPV OR sIPV OR "Sabin inactivated poliovirus vaccine") AND ("Booster" OR "adult vaccination" OR "revaccination") AND ("Safety"[Mesh] OR "adverse events" OR "reactogenicity" OR "tolerability") AND ("Randomized Controlled Trial"[Publication Type] OR "randomized controlled trial" OR RCT)** | **MeSH terms + keywords** | **78** |
| **Cochrane Library** | **('inactivated poliovirus vaccine' OR 'Sabin inactivated poliovirus vaccine' OR sipv OR ipv OR 'polio vaccine') AND (booster OR revaccination OR 'adult vaccination') AND (safety OR 'adverse event' OR 'adverse events' OR reactogenicity OR tolerability) AND (randomized OR randomised OR 'randomized controlled trial' OR rct)** | **Keywords (Title/Abstract/Keywords)** | **155** |
| **Embase** | **('inactivated poliovirus vaccine' OR 'Sabin inactivated poliovirus vaccine' OR sipv OR ipv OR 'polio vaccine') AND (booster OR revaccination OR 'adult vaccination') AND (safety OR 'adverse event' OR 'adverse events' OR reactogenicity OR tolerability) AND (randomized OR randomised OR 'randomized controlled trial' OR rct)** | **Keywords** | **230** |
| **Scopus** | **TITLE-ABS-KEY("inactivated poliovirus vaccine" OR "Sabin inactivated poliovirus vaccine" OR sipv OR ipv OR "polio vaccine") AND TITLE-ABS-KEY(booster OR revaccination OR "adult vaccination") AND TITLE-ABS-KEY(safety OR "adverse event" OR "adverse events" OR reactogenicity OR tolerability) AND TITLE-ABS-KEY(randomized OR randomised OR "randomized controlled trial" OR rct)** | **Title, Abstract, Keywords** | **142** |

**Supplementary Table 1.** Full search strategy.

| Outcomes | No. of participants (studies) | Effect estimates (95% CI) | Inconsistency | Indirectness | Imprecision | Publication Bias | Quality |
| --- | --- | --- | --- | --- | --- | --- | --- |
| Any Pain | **110 (3)** | **RR 1.57 [0.98, 2.50]** | **Not serious** | **Not serious** | **Serious** | **N/A** | **Low** |
| Erythema | **330 (4)** | **RR 1.54 [0.64, 3.69]** | **Not serious** | **Not serious** | **Minor** | **N/A** | **Moderate** |
| Induration | **70 (2)** | **RR 0.33 [0.04, 3.06]** | **Not serious** | **Not serious** | **Serious** | **N/A** | **Very Low** |
| Fever | **340 (4)** | **RR 0.96 [0.39, 2.33]** | **Not serious** | **Not serious** | **Minor** | **N/A** | **Moderate** |
| Headache | **70 (2)** | **RR 0.70 [0.21, 2.37]** | **Not serious** | **Not serious** | **Serious** | **N/A** | **Low** |
| Malaise | **130 (3)** | **RR 0.75 [0.17, 3.27]** | **Not serious** | **Not serious** | **Serious** | **N/A** | **Low** |
| Moderate Pain | **270 (3)** | **RR 3.28 [0.69, 15.53]** | **Not serious** | **Not serious** | **Serious** | **N/A** | **Low** |
| Mild Pain | **80 (2)** | **RR 1.98 [0.90, 4.40]** | **Not serious** | **Not serious** | **Serious** | **N/A** | **Low** |

**Supplementary Table 2.** Certainty of evidence (GRADE assessment).

**Supplementary Figures**

**Supplementary Figure 1.** Malaise, sIPV vs control (RR).
**Supplementary Figure 2.** Moderate pain, sIPV vs control (RR).
**Supplementary Figure 3.** Mild pain, sIPV vs control (RR).

**Supplementary Figure 4.** Sensitivity analysis for moderate pain, sIPV vs control (RR).

**Supplementary Figure 5.** Sensitivity analysis for malaise, sIPV vs control (RR).

**Supplementary Figure 6.** Sensitivity analysis for fever, sIPV vs control (RR).

**Supplementary Figure 7.** Sensitivity analysis for erythema, sIPV vs control (RR).

**Supplementary Figure 8.** Sensitivity analysis for any pain, sIPV vs control (RR).

**Supplementary Figure 9.** Funnel plot assessing publication bias for all outcomes.

**Supplementary Tables**

**Supplementary Table 1.** Full search strategy.
**Supplementary Table 2.** Certainty of evidence (GRADE assessment).
